# Influenza Virus A Infection of Human Monocyte and Macrophage Subpopulations Reveals Increased Susceptibility Associated with Cell Differentiation

**DOI:** 10.1371/journal.pone.0029443

**Published:** 2012-01-04

**Authors:** Marieke A. Hoeve, Anthony A. Nash, David Jackson, Richard E. Randall, Ian Dransfield

**Affiliations:** 1 MRC Centre for Inflammation and Research, Queen's Medical Research Institute, University of Edinburgh, Edinburgh, United Kingdom; 2 Centre for Infectious Diseases, The Roslin Institute and Royal (Dick) School of Veterinary Studies, University of Edinburgh, Edinburgh, United Kingdom; 3 Biomedical Sciences Research Complex, University of St. Andrews, St. Andrews, United Kingdom; University of Ottawa, Canada

## Abstract

Influenza virus infection accounts for significant morbidity and mortality world-wide. Interactions of the virus with host cells, particularly those of the macrophage lineage, are thought to contribute to various pathological changes associated with poor patient outcome. Development of new strategies to treat disease therefore requires a detailed understanding of the impact of virus infection upon cellular responses. Here we report that human blood-derived monocytes could be readily infected with the H3N2 influenza virus A/Udorn/72 (Udorn), irrespective of their phenotype (CD14^++^/CD16^−^, CD14^++^/CD16^+^ or CD14^dim^CD16^++^), as determined by multi-colour flow cytometry for viral haemagglutinin (HA) expression and cell surface markers 8–16 hours post infection. Monocytes are relatively resistant to influenza-induced cell death early in infection, as approximately 20% of cells showed influenza-induced caspase-dependent apoptosis. Infection of monocytes with Udorn also induced the release of IL-6, IL-8, TNFα and IP-10, suggesting that NS1 protein of Udorn does not (effectively) inhibit this host defence response in human monocytes. Comparative analysis of human monocyte-derived macrophages (Mph) demonstrated greater susceptibility to human influenza virus than monocytes, with the majority of both pro-inflammatory Mph1 and anti-inflammatory/regulatory Mph2 cells expressing viral HA after infection with Udorn. Influenza infection of macrophages also induced cytokine and chemokine production. However, both Mph1 and Mph2 phenotypes released comparable amounts of TNFα, IL-12p40 and IP-10 after infection with H3N2, in marked contrast to differential responses to LPS-stimulation. In addition, we found that influenza virus infection augmented the capacity of poorly phagocytic Mph1 cells to phagocytose apoptotic cells by a mechanism that was independent of either IL-10 or the Mer receptor tyrosine kinase/Protein S pathway. In summary, our data reveal that influenza virus infection of human macrophages causes functional alterations that may impact on the process of resolution of inflammation, with implications for viral clearance and lung pathology.

## Introduction

Seasonal influenza infection annually affects about 10% of the population. Although in most patients the infection is self-limiting and resolves over time, the virus can also cause severe viral pneumonia, secondary bacterial infections, respiratory failure and death, particularly in older patients or in the very young. Each year one million influenza-associated deaths are attributed to seasonal influenza strains [1], [2], [3], while the emergence of pandemic strains poses an even greater health threat. Current antiviral strategies for treatment include inhibitors of the influenza virus M2 ion channels (amantadine and rimantadine) or inhibition of neuraminidase activity (neuraminidase inhibitors) to limit viral spread [4]. However, there is a growing appreciation that innate and adaptive immune regulatory mechanisms are pivotal determinants of disease outcome [5]. In particular, macrophages and their products (cytokines and chemokines) are thought to play a key role in controlling infection and thus may represent targets for new, effective therapeutic intervention strategies for treatment of influenza virus infection. A detailed understanding of the interplay between virus and macrophages and their potential impact upon processes that are relevant to disease pathogenesis would be required to utilize regulation of immune pathways to control influenza disease.

Alveolar macrophages represent the predominant phagocyte population present within the lung in the absence of infection. They have an important homeostatic function, with a relatively low capacity for phagocytosis and production of inflammatory cytokines in the absence of activation. Following viral infection of alveolar macrophages, their activation can dramatically alter cytokine and growth factor production [6], [7]. In addition, infection of respiratory airway epithelial cells (AEC) with influenza virus triggers release of cytokines and chemokines (including IP-10, IFNβ, RANTES and IL-6) [8] that promote the recruitment of blood-derived inflammatory cells, including neutrophils and monocytes [9], [10], [11]. Alveolar macrophage activation together with initiation of inflammatory cell recruitment contributes to virus-induced pathology and mortality [12]. Recently, it has been reported that H5N1 highly pathogenic avian influenza virus induced production of very high levels of TNFα and IFNβ in monocyte-derived macrophages [13], raising the possibility that high levels of cytokines produced by macrophages were associated with excessive disease pathology. Consistent with this, mice lacking TNFα and IL-1 receptors have reduced inflammatory responses following infection [10], while macrophages lacking critical counter-regulatory signalling pathways exhibited more severe lung pathology [14]. Surprisingly, IL-10, which might be predicted to have anti-inflammatory activity, was found to reduce development of protective immunity in mice [15], indicating an additional level of complexity in the cytokine regulation of immunity to virus infection.

Influenza infection has been shown to trigger apoptosis in MDCK and HeLa cells [16], [17] and in lung airway epithelial cells [18], [19] and has been suggested to be important for virus clearance *in vivo* [20], e.g. by directly inhibiting the production of new virus [21] and triggering the release of pro-inflammatory cytokines by bystander cells, thereby limiting both spread of infection and the potential for promotion of inflammation. In addition, apoptotic cell clearance has the potential to actively counter the production of pro-inflammatory cytokine production and to initiate release of immune-regulatory mediators (e.g. IL-10 and TGFβ) that can direct the resolution of inflammation [22]. Moreover, specific removal of apoptotic cells by tissue phagocytes limits the potential of infected cells to undergo secondary necrosis and release pro-inflammatory stimuli (e.g. HMGB1, HSPs and formylated peptides), proteases and oxidant species that could cause tissue damage. However, evidence from animal models of influenza infection suggests that reduction of AEC apoptosis can ameliorate pathology and increases host survival [18], raising the possibility that the net outcome of the responses may be dictated by host-, cell- and/or pathogen specific factors [23].

While the impact of infection with influenza virus has been reasonably well studied in murine models both *in vitro* and *in vivo* [24], the susceptibility and functional responses of human innate immune cells to influenza virus infection remains relatively ill-defined. Specifically, the phenotype of human blood-derived monocytes and macrophages infected with influenza virus has not been reported and the extent of infectivity has not been assessed. Here we address this by assessing human monocyte and monocyte-derived macrophage function in the context of influenza virus infection. In addition, we provide the first analysis of the permissiveness of different subpopulations of human blood-derived monocytes and well-defined monocyte-derived macrophage phenotypes to human influenza virus (Udorn; H3N2). Monocytes and macrophages became infected with Udorn virus regardless of cell phenotype and were found to be relatively resistant to caspase-dependent influenza-induced cell death. In addition to inducing cytokine and chemokine release by monocytes and macrophages, influenza virus infection caused increased macrophage capacity for phagocytosis of apoptotic cells. Together, these findings increase our understanding of the interplay between human influenza virus and host cell responsiveness, which may ultimately lead to new strategies to modulate disease outcome.

## Results

### Influenza infection of human blood monocytes

**Figure 1:**
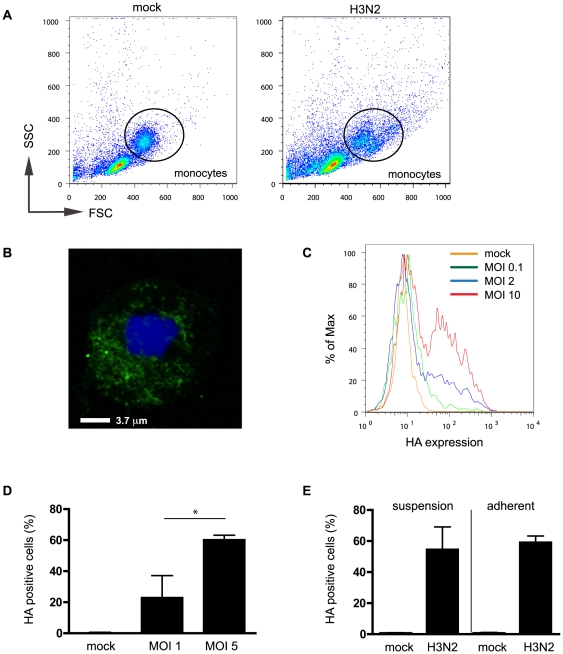
Influenza infection of human monocytes. Representative examples of laser scatter histograms of uninfected and Udorn-infected MNC cultured in Teflon pots (**A**) and of HA expression in human monocytes as assessed by confocal microscopy (**B**) and flow cytometry (**C**). (**D**) HA expression shows dose-dependency. (**E**) Plate adherence is not required for HA expression by infected monocytes as results are similar for cells infected in Teflon pots and tissue culture plate. Bars represent means of 3–6 donors ± SD. Unless otherwise indicated, data represent 8 h p.i. and MOI = 2. *: p<0.05.

The capacity of human monocytes to become infected with an H3N2 seasonal human influenza virus (Udorn) was determined by assessment of viral haemagglutinin (HA) expression using both flow cytometry and confocal microscopy. These experiments were performed with mononuclear cells (MNC) in suspension culture using Teflon pots to prevent adherence of monocytes to tissue culture plastic, which has been shown to result in activation, including induction of early response genes (c-fos) and cytokines [25]. Incubation of MNC in the presence of Udorn (but not UV-inactivated virus) caused a change in forward and side scatter of cells as assessed by flow cytometry (Figure 1A), suggesting that Udorn infection caused changes in cell size/granularity. However, there was no direct correlation between altered laser scatter profiles and viral infection, since HA was also expressed by non-shifted cells. Monocytes were found to express HA both intracellularly by confocal microscopy 8 h post infection (p.i.) (Figure 1B) as well as at the cell surface, as determined with flow cytometry (Figure 1C). In contrast, lymphocytes present within MNC populations incubated with Udorn, or purified CD3^+ve^ cells (isolated using immunomagnetic selection) did not express HA as assessed by flow cytometry (data not shown). Infection of monocytes with Udorn was not dependent on the presence of lymphocytes, as monocytes isolated using CD14^+^ immunomagnetic selection incubated with Udorn (MOI = 2) showed a similar HA expression at 8 h p.i. to gated monocytes in MNC samples (48.7±5.5 (n = 3) and 47.1±6.5 (n = 9), respectively; data not shown). The extent of HA expression was related to virus dose (Figure 1C and Figure 1D; MOI = 1 vs MOI = 5: p<0.05) and showed a strict time dependency (data not shown). The infection was productive, with virion release in a virus-dose and time-dependent manner (41.7±9.5 (MOI = 0.5) and 110.2±22.8 (MOI = 2) in PFU/ml ×10^3^ for 3 donors (8 h p.i.), and data not shown). Finally, we tested whether adherence of monocytes to tissue culture plastic would impact upon HA expression. Surprisingly, we did not find a significant difference in infectivity when monocytes were cultured in suspension or adherent to tissue culture plates (Figure 1E), indicating that adhesion-induced activation does not confer protection against infection.

### Infection of different human monocyte subsets with influenza virus

**Figure 2:**
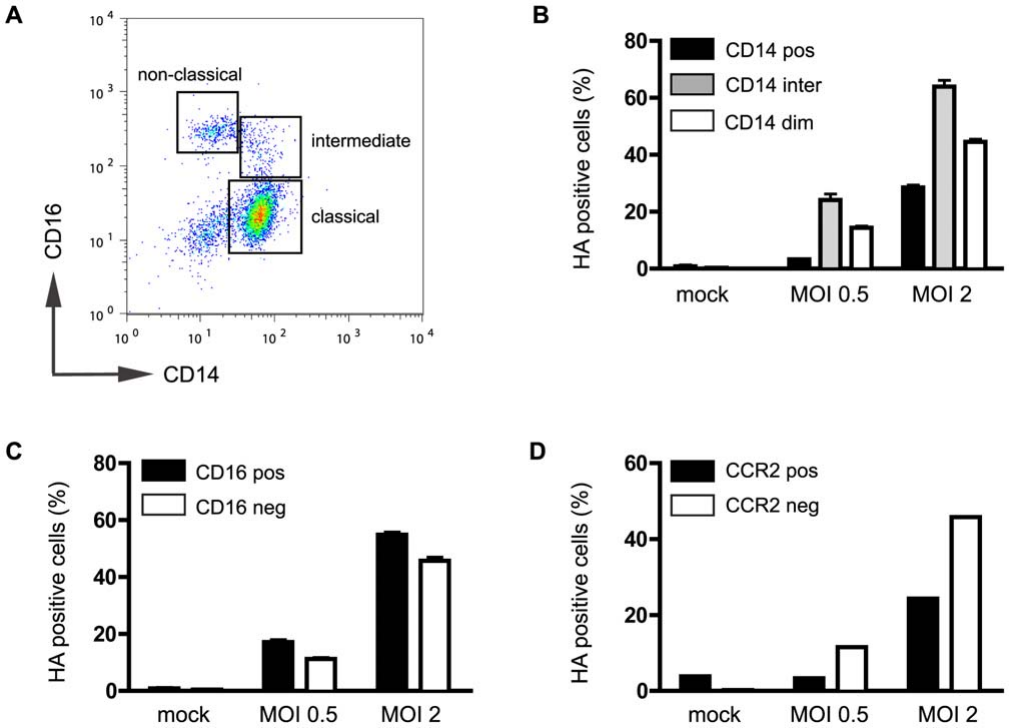
Influenza infection of human monocyte subsets. (**A**) Double colour flow cytometry with anti-CD14 and CD16 antibodies allows detection of various monocyte populations within human MNC populations (monocytes gated as shown in Figure 1). The CD14 (**B**), CD16 (**C**) and CCR2 (**D**) positive and negative subpopulations are all susceptible to infection with Udorn, as HA^+ve^ cells could be detected in each of the subpopulations (MOI = 2, 8 h p.i.). Histogram and graphs are representative examples of 3 independent experiments.

We next assessed whether monocyte subsets present in the circulation differ in their capacity for influenza virus infection. Expression of viral HA on monocyte subsets was assessed using a combination of CD14, CD16 and CCR2 (CD192) monoclonal antibodies. Populations of monocytes with CD14^++^/CD16^−^ (classical), CD14^++^/CD16^+^ (intermediate) and CD14^dim^CD16^++^ (non-classical) expression profiles were readily detected in uninfected (Figure 2A) and Udorn-infected MNC preparations (Figure 2B–D), with CD14 positive, intermediate or dim, CD16 positive or negative and CCR2 positive or negative cells present. There were no changes in the percentages of CD16 positive and CD16 negative, or CCR2 positive and CCR2 negative monocyte subpopulations observed following infection with Udorn (Table 1). Although we did observe a decreased percentage of CD14 positive cells and consequently an increased percentage of CD14 dim cells 8 h after infection, this was not statistically significant. These experiments demonstrated that viral infectivity was not limited to any specific monocyte subset. To dissect this further we performed a flow cytometry-based assessment of sialic acid containing glycoconjugates on monocytes and found similar staining by *Sambucus nigra agglutinin* lectin (SNA I, which recognizes α(2,6)-linked sialic acids) to classical (CD14^++^/CD16^−^) and non-classical (CD14^dim^/CD16^++^) monocytes (fluorescence ratio non-classical:classical monocytes = 1.06±0.1 (n = 5); data not shown), while cell staining with *Maackia amurensis lectin II* (Mal II, which binds to α(2,3)-linked sialic acids) was ∼2-fold stronger on non-classical monocytes compared to classical monocytes (fluorescence ratio non-classical:classical monocytes = 1.88±0.3 (n = 5); data not shown).

**Table 1:** Influenza infection of human monocyte subsets.

	mock	MOI 0.5	MOI 2
**CD14 pos**	66.9±9.5	56.9±11.6	41.8±15.3
**CD14 inter**	14.8±5.1	11.9±2.2	14.2±2.7
**CD14 neg**	16.2±5.8	28.9±12.4	41.3±16.6
**CD16 pos**	9.4±1.8	12.4±1.7	11.5±2.2
**CD16 neg**	90.4±1.7	87.6±1.6	88.5±2.2
**CCR2 pos**	5.4±1.6	8.5±1.3	6.3±1.7
**CCR2 neg**	94.6±1.6	91.5±1.3	93.7±1.8

The relative percentages of the various monocyte subsets in human MNC were not affected by infection with Udorn virus (8 h p.i.) compared to mock-infection. Data represent 3 independent experiments (mean ±SEM).

### Influenza infection of human monocyte-derived macrophages

**Figure 3:**
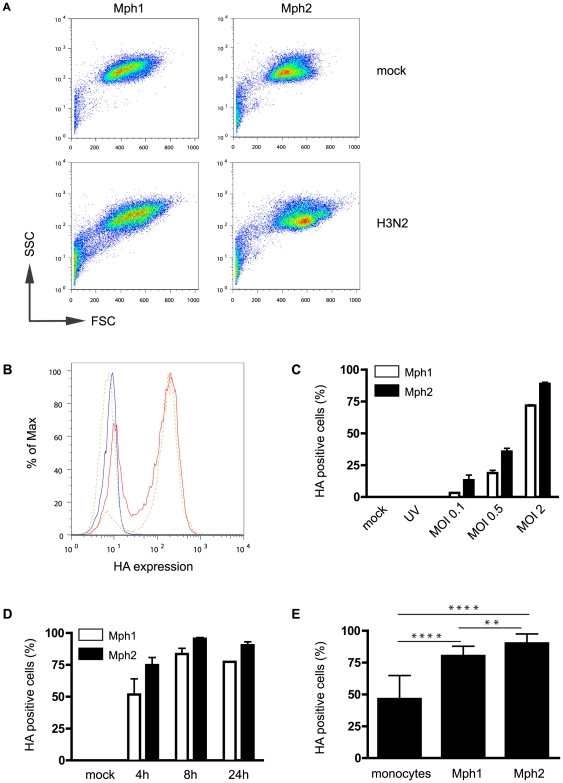
Influenza infection of human monocyte-derived macrophages. (**A**) Laser scatter histograms of mock- and Udorn-infected Mph1 and Mph2 cells. (**B**) HA expression by infected live Mph1 (dashed green line) and Mph2 (dashed blue line) cells over mock treated Mph1 (solid orange line) and Mph2 (solid red line) as assessed by flow cytometry. HA expression shows dose (**C**) and time (**D**) dependency in both macrophage subsets. (**E**) Mph1 and Mph2 show a higher HA^+ve^ cell percentage than monocytes. Flow plots are representative examples of 6 independent experiments. Bars represent means of 4–9 experiments ± SD. Unless otherwise indicated, data represent 8 h p.i. and MOI = 2. **: p<0.01; ****: p<0.0001.

To further explore the effects of monocyte differentiation status upon susceptibility to infection with influenza we next treated CD14^+ve^ monocytes with GM-CSF or M-CSF to drive differentiation towards Mph1 (macrophages with pro-inflammatory characteristics) or Mph2 (macrophages with anti-inflammatory characteristics), respectively, as we reported previously [26]. These experiments revealed that Udorn was capable of infecting human blood monocyte-derived macrophages (Mph) irrespective of their functional properties. Flow cytometric analyisis of Mph1 and Mph2 (Figure 3A) indicated that both Mph phenotypes expressed HA (Figure 3B) in a dose- (Figure 3C) and time- (Figure 3D) dependent manner, following incubation with live, but not UV-inactivated Udorn virus (Figure 3C). In a comparative analysis of the proportion of HA expressing cells, Mph showed increased infectivity (∼1.8 fold) when compared with MNC incubated with the same viral dose (Figure 3E; p<0.0001 for Mph1 and Mph2). Of note, two epithelial cell lineages, A549 and MDCK cells, also showed increased proportions of HA expression when compared to monocytes (∼1.7 fold and ∼2.3 fold, respectively, with MOI = 2 at 8 h p.i.; data not shown). Moreover, with a high virus dose we obtained near 100% infectivity for Mph1 (97%), Mph2 (96%), A549 (91%) and MDCK (94%) cells, but not monocytes (61%) (MOI = 10, 8 h p.i.; data not shown). Western blot analysis confirmed influenza virus replication in human monocyte-derived Mph, as we detected viral NS1, NP, HA and M1 proteins in cell lysates 8 h post infection with MOI = 2 and MOI = 5 (Figure S1). These influenza proteins were not expressed in mock-infected, UV-treated virus infected, or Udorn-infected Mph treated with the protein synthesis inhibitor cycloheximide, while β-actin was readily detected in all samples (Figure S1). Use of high MOI induced high levels of apoptosis in monocytes and Mph (see below), thus subsequent infection experiments were performed with viral doses ranging from MOI = 0.1 to 5. Interestingly, in a series of paired experiments comparing Mph1 and Mph2 derived from the same donors, the percentage of HA-positive cells was significantly higher in Mph2 than Mph1 samples when using a dose of MOI = 2 for 8 h (Fig. 3E: 80.09±2 vs 90.01±2; p<0.01; n = 5 donors). Yet, virion release (which showed a virus-dose and time dependency) was greater for Mph1 than Mph2 (representative example in triplicate: 157.5±17.5 and 306.3±38.1 (Mph1) versus 75±5 and 176.3±11.3 (Mph2) in PFU/ml ×10^3^ for MOI = 2, 8 h and 24 h p.i., respectively (data not shown)).

### Human monocytes and monocyte-derived macrophages are resistant to influenza-induced apoptosis

**Figure 4:**
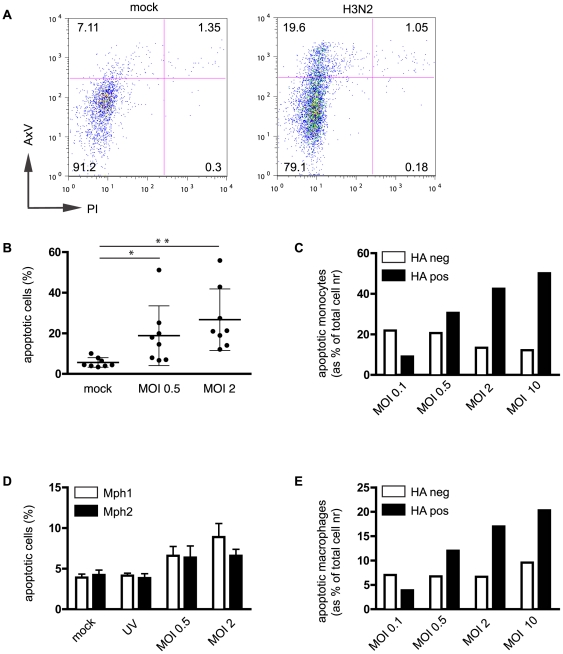
Influenza infection induces apoptosis in human monocytes and monocyte-derived macrophages. (**A**) Influenza infection of MNC increases the percentage of apoptotic (AxV^+^/PI^−^) but not necrotic (AxV^+^/PI^+^) monocytes, as assessed by flow cytometry (MOI = 2). Infectious Udorn virus, but not UV-inactivated virus (UV) induces apoptosis in human MNC (**B**) and human macrophages (both Mph1 & Mph2) (**D**) in a dose dependent manner (**B** and **D**), with increasing numbers of apoptotic cells expressing HA with increasing virus dose in both cell types (MNC in **C**, and Mph1 in **E**). Bars represent means of 8 (**B**) or 3 (**D**) experiments ±SD, or representative examples (**A,C,E**). All experiments are 8 h p.i. *: p<0.05; **: p<0.01.

To assess the relationship between infection with influenza virus and the induction of apoptosis of human monocytes and Mph we determined the percentage of apoptotic cells (AxV^+^/PI^−^) and necrotic cells (AxV^+^/PI^+^) by flow cytometry (Figure 4A). We observed reproducible induction of low percentages of apoptosis, in the absence of detectable necrosis, following Udorn-infection of monocytes and Mph for 8 h (Figure 4A and data not shown). Induction of apoptosis was dependent on the extent of infection in both monocytes and Mph, displaying a close relation to virus MOI (Figure 4B and 4D, respectively) and infection time (data not shown). Infection with a high virus load did induce relatively high levels of AxV^+ve^ cells (Figure 4C and 4E, and data not shown). Multi-colour flow cytometry revealed that apoptotic cells were present in both the HA^+ve^ and HA^−ve^ population in monocyte- (Figure 4C) and Mph cultures (Figure 4E), with the relative contribution of HA^+ve^ cells to the AxV^+ve^ pool increasing with increasing virus dose (Figure 4C and 4E).

### Caspase-dependent induction of HA expression in influenza virus infected human monocytes

**Figure 5:**
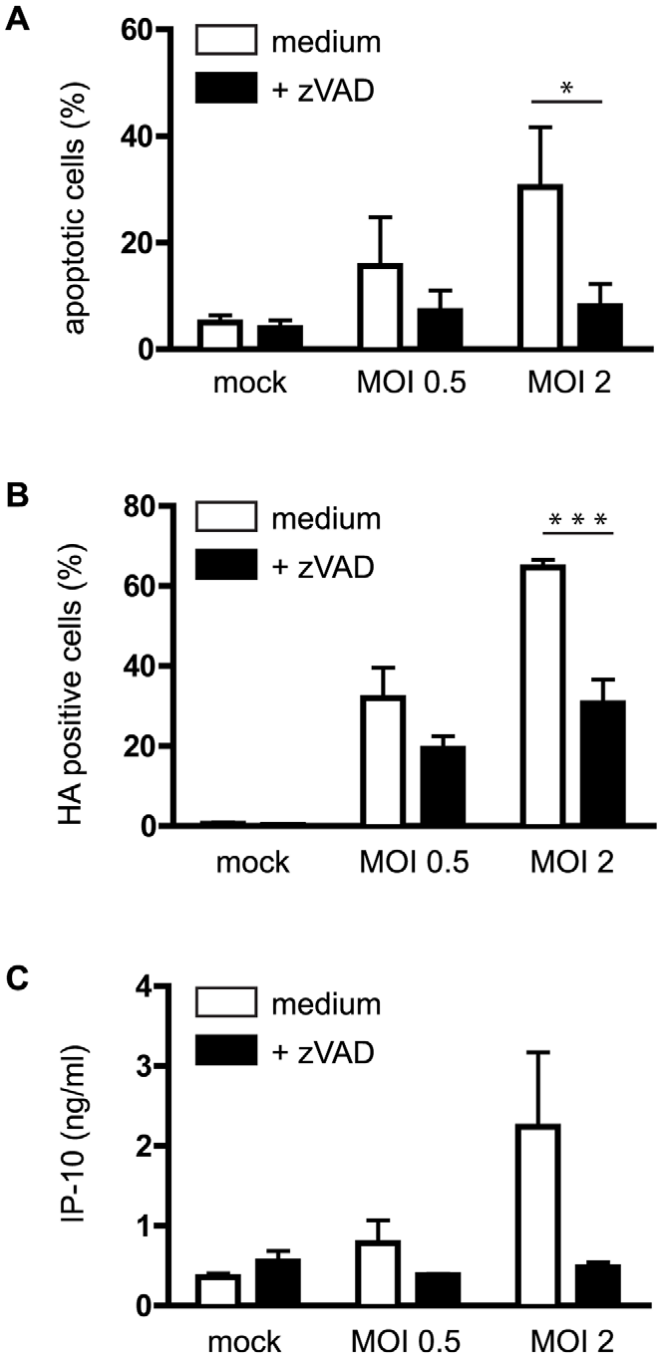
Caspase dependency of influenza-induced apoptosis, HA expression and IP-10 production in human monocytes. Apoptosis (**A**), HA expression (**B**) and IP-10 secretion (**C**) following infection of human monocytes with influenza virus (8 h p.i.) are dependent on caspase activity, as the pan-caspase inhibitor zVAD reduces apoptosis, HA positivity and IP-10 release. Bars represent mean ± SD of 3 independent experiments. *: p<0.05; ***: p<0.001.

Given the association between virus infection and induction of apoptosis shown in Figure 4, we next sought to determine the impact of inhibiting caspase enzyme activity upon expression of virus HA protein and induction of apoptosis in monocytes. As would be expected, the proportion of monocytes that were induced into apoptosis following incubation with Udorn was greatly reduced by the pan-caspase inbibitor zVAD, with proportions of annexin V^+ve^ cells comparable to those of mock-infected cells (∼8% for Udorn-infected cells and ∼4% for mock-infected cells (Figure 5A; reduction with MOI = 2: p<0.05). The inhibition of apoptosis was not overcome by increasing virus dose (Figure 5A), suggesting that zVAD inhibition of caspase activity was effective. Of note, zVAD did not interfere with virus attachment to the cell or block virus replication, as Western blot analysis demonstrated that zVAD treatment did not preclude expression of influenza proteins NS1, HA, NP and M1 in Mph 8 h pi with MOI = 5 (Figure S1). In contrast to the almost complete inhibition of Udorn-induced apoptosis in infected monocytes treated with zVAD, expression of HA was still readily detectable on zVAD-treated monocytes 8 h p.i. The proportion of HA^+ve^ cells was reduced by ∼50% in the presence of zVAD after infection with MOI = 2 (Figure 5B; p<0.001) and comparison of the level of surface expression (mean fluorescent intensity (MFI) of staining) of HA on gated monocytes revealed a two-fold MFI reduction after zVAD treatment: 64.8±8.2% reduction of MFI with MOI = 0.5 (n = 3) and 70.4±5.4% reduction with MOI = 2 (n = 3) (data not shown). It is worth noting that previous reports have shown that while early events in the viral life cycle such as virus entry, genome release, and transcription and translation do not rely on caspase activity, caspases are required for generation of progeny virus [27], likely by enabling export of ribonucleoprotein complexes from the nucleus to the cytoplasm of infected cells [28].

### Cytokine release by influenza infected human monocyte- and monocyte-derived macrophage subsets

**Figure 6:**
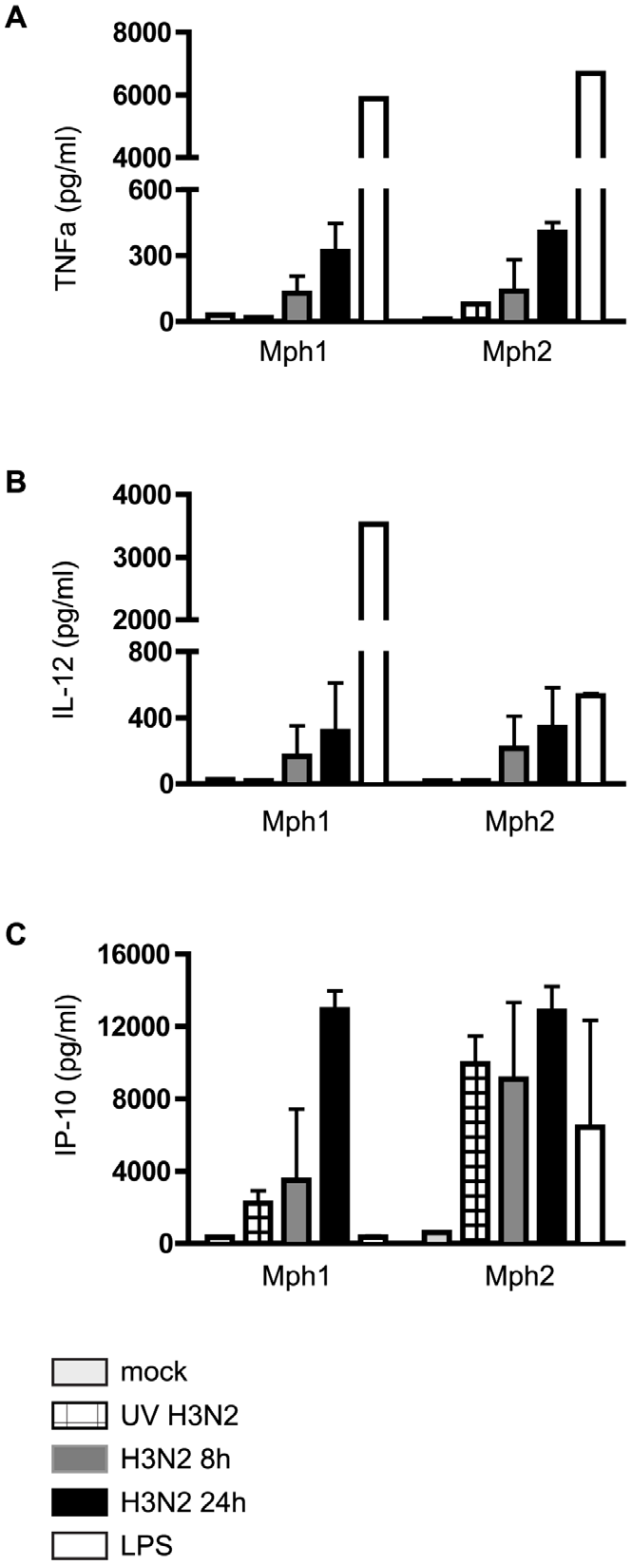
Influenza infection of human monocyte-derived macrophages induces cytokine/chemokine release. Influenza infection induces secretion of TNFα (**A**), IL-12p40 (**B**) and IP-10 (**C**) by human Mph1 and Mph2 cells, as assessed by ELISA of culture supernatants of mock-infected, UV-inactivated (8 h p.i.) and infectious Udorn virus-infected cells (MOI = 2, 8 and 24 h p.i.). Data are mean protein levels ± SD; n = 4–8 experiments.

We have previously shown that human monocyte-derived Mph subsets show differential responses to TLR-4 ligand LPS, with Mph1 secreting IL-12 but no IL-10, and Mph2 producing IL-10 but low levels of IL-12, following LPS stimulation [26], [29]. We therefore determined the impact of influenza virus infection upon cytokine/chemokine production profiles in both Mph1 and Mph2 by ELISA. We found that Udorn-infection induced comparable TNFα (Figure 6A), IL-12p40 (Figure 6B), IP-10 (Figure 6C) and IL-6 (data not shown) levels in both Mph1 and Mph2. Influenza-induced TNFα and IL-6 (Mph1 & Mph2) and IL-12 (Mph1) levels were 5–20 fold lower than those induced by LPS, although induction of IP-10 by influenza was similar to LPS-induced IP-10 (Mph1 and Mph2, respectively). Induction of TNFα, IL-12 and IL-6 required the presence of live virus, as UV-inactivated Udorn did not trigger secretion of these cytokines. In contrast, release of IP-10 did not show this requirement, as IP-10 was also secreted after incubation with UV-inactivated virus, especially in Mph2 (Figure 6C). Secretion of TNFα, IL-12p40, IP-10 and IL-6 showed a dependency upon infection time (Figure 6 and data not shown) and upon virus dose (data not shown). IL-8 production by Mph was constitutive in mock-infected Mph1 and Mph2 cells, with no or only a slight increase due to incubation with live- or UV-inactivated virus for most donors (data not shown). We did not detect IL-10 release in response to Udorn-infection in either Mph1 or Mph2 of any donor (n = 6; data not shown). In contrast, Mph2 of these donors produced 2–7 ng/ml IL-10 following LPS stimulation ([26] and data not shown). IL-33 was not produced by either Mph subset 8–16 h after mock-infection, Udorn-infection or LPS stimulation (data not shown). Similar to the findings for Mph, infection of human monocytes with infectious Udorn specifically induced secretion of TNFα, IL-6, IL-8 and IP-10, levels of which were reduced to background levels when cells were treated with the caspase inhibitor zVAD (Figure 5C and data not shown). However, influenza infection of monocytes did not induce production of IL-10, IL-12 or IL-33 (data not shown).

### Influenza infection of human monocyte-derived macrophages increases their capacity to phagocytose apoptotic cells

**Figure 7:**
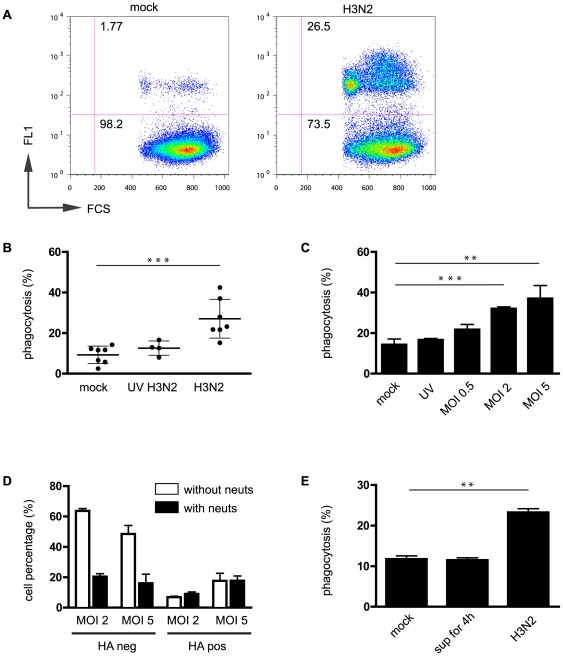
Influenza infection of human monocyte-derived macrophages increases their capacity to phagocytose apoptotic cells. (**A** and **B**) Infection of human Mph1 cells with Udorn virus increases phagocytosis of apoptotic neutrophils as determined with flow cytometry using green fluorescent neutrophils. This increase, which is virus-dose dependent (**C**), was not seen when using UV-inactivated virus (**B** and **C**) or UV-inactivated supernatants that were harvested 12 h p.i. of Mph1 and added 4 h prior to phagocytosis (**E**). (**D**) Green neutrophils (neuts) were ingested by both HA negative and HA positive Mph1 cells. (**B**) Depicts 7 experiments ± SD, while the other panels depict representative examples performed in triplicate (mean ± SD). Unless otherwise indicated, cells were infected for 16 h with MOI = 1. **: p<0.01; ***: p<0.001.

The impact of virus infection on the function of Mph was next investigated by examining the capacity for phagocytosis of two distinct targets: latex microspheres (1 µm) and apoptotic neutrophils (∼7 µm). Influenza infection did not affect uptake of microspheres by either Mph population, even at high virus MOI and high bead to macrophage ratios (percent phagocytosis at bead to macrophage ratio of 10∶1; mock-infected *vs* H3N2-infected was 63.5±1.2 *vs* 64.7±3.7 for Mph1, and 75±2.3 *vs* 78.6±2.5 for Mph2 (MOI = 1; t = 16 h) (representative experiment performed in triplicate; data not shown). In contrast, there was a significant increase in the proportion of Mph1 able to phagocytose apoptotic neutrophils (Figure 7A) after infection with infectious Udorn virus (p<0.001) but not UV-inactivated virus (Figure 7B), with an average fold-increase of 4.1±1.3 (n = 7). The increase in the capacity for phagocytosis of neutrophils by infected Mph1 showed a virus-dose dependency (Figure 7C). Similar results were obtained when using apoptotic airway epithelial cell lines (A549 and Beas 2B cells) as targets (data not shown). In contrast, for Mph2 cells there was no influenza-induced increase in phagocytosis of apoptotic neutrophils (average fold increase for Mph2 was 0.92±0.2 (n = 5)) or epithelial cells (data not shown). Multi-laser flow cytometry revealed that the fluorescent neutrophils were ingested by HA^+ve^ as well as HA^−ve^ Udorn-infected Mph1 cells (Figure 7D) and Mph2 cells (data not shown), with the percentage of ingested neutrophils in HA^+ve^ Mph1 cells (but not HA^−ve^ Mph1 cells) increasing with increasing virus dose (Figure 7D).

The influenza infection-associated increase of phagocytosis could not be mimicked with culture supernatant of Udorn-infected Mph, as addition of (UV-inactivated) supernatants collected 12 h and 14 h p.i. of Mph1 or Mph2 (MOI = 1) onto non-infected Mph 2 h or 4 h before phagocytosis did not affect subsequent uptake of apoptotic neutrophils (Figure 7E and data not shown). The uptake of apoptotic neutrophils by Mph was not affected by addition of rIL-10 or Protein S 30 min prior to and during phagocytosis (data not shown). Moreover, the influenza-induced increase of phagocytosis of apoptotic neutrophils was not dependent on the Mer receptor tyrosine kinase (Mertk) pathway, as addition of an anti-Mer blocking antibody did not inhibit phagocytosis of apoptotic neutrophils, either in mock-infected or in Udorn-infected Mph (percent phagocytosis by Udorn-infected macrophages +/− aMer: 21.2±1.2 *vs* 20.8±3 for Mph1, and 56.7±2.5 *vs* 54.1±1 for Mph2 (representative experiment performed in triplicate; data not shown)).

## Discussion

We have established an influenza infection model using human blood monocytes and monocyte-derived macrophages (Mph) to gain a better understanding of the impact of virus infection upon cellular responses in man. Monocytes could be infected with influenza H3N2 strain A/Udorn/72 (Udorn), with ∼50% of cells expressing viral HA 8–16 h after infection (MOI = 2). In contrast, lymphocytes within MNC samples or as purified CD3^+ve^ cells did not express HA, indicating a leukocyte cell type specific infectivity profile for Udorn virus.

It is well established that circulating human monocytes can be divided into distinct subpopulations based on specific cell surface receptor profiles, including CD14 and CD16 [30], [31], [32], [33]. These ‘classical’ (CD14^++^/CD16^−^), ‘intermediate’ (CD14^++^/CD16^+^) and ‘non-classical’ (CD14^dim^/CD16^++^) monocytes [34], [35], [36] display distinct functional properties *in vitro* and *in vivo* [37], [38], with classical monocytes suggested to be pro-inflammatory, while the non-classical cells patrol tissues and reportedly selectively recognize nucleic acid [34]. Multi-colour flow cytometry revealed that monocyte subsets expressed comparable levels of viral HA 8 h p.i., suggesting that influenza permissiveness is not restricted to specific monocyte populations, nor dependent on CD14, as has been reported for influenza-induced cytokine/chemokine release [39]. Furthermore, although we observed a reduced percentage of CD14^+ve^ cells (Table 1), this difference was not statistically significant and likely due to loss of CD14 expression following influenza-induced apoptosis rather than a specific influenza-mediated down-regulation of CD14, as previously suggested [39].

Assessment of lectin expression supports this notion of non-restricted permissiveness, as classical (CD14^++^/CD16^−^) and non-classical (CD14^dim^/CD16^++^) monocytes showed similar staining by SNA I. This lectin preferentially recognizes α(2,6)-linked sialic acids [40], while Udorn virus exhibits preferential sialic acid cleavage activity for α(2,6)-linked sialosides [41]. In contrast, cell staining with MalII (which binds to α(2,3)-linked sialic acids) was ∼2-fold stronger on non-classical monocytes compared to classical monocytes, which potentially predisposes the former to infection with human-, and especially avian influenza viruses. However, a high-throughput neuraminidase substrate screen of a large pool of influenza viruses of varied origin showed that sialic acid substrate preference and the degree of sialic acid hydrolysis activity vary significantly with virus strain- and NA type [41], suggesting that extrapolating our findings to other influenza viruses should be undertaken with caution. We conclude that even though their functions are distinct, monocytes with either a pro-inflammatory (‘classical’) or anti-inflammatory (‘non-classical’) phenotype show a comparable susceptibility to influenza A/Udorn/72 virus.

No difference in influenza virus infectivity of adherent versus suspension-cultured monocytes was found, suggesting that adhesion-induced monocyte activation [25] does not protect against infection. Indeed, differentiation towards Mph increased susceptibility to influenza nearly 2-fold, with ∼80–90% of monocyte-derived Mph expressing HA 8–16 h p.i. with Udorn virus. Previously reported rapid expression of HA, NP and M1 protein and cytokine release in GM-CSF-treated, A/Puerto Rico/8/34 virus-infected monocytes suggests that cell differentiation readily confers susceptibility to influenza [42]. Altered surface glycosylation associated with Mph differentiation may facilitate virus association. Induction of expression and activity of endogenous neuraminidase 1 associated with monocyte differentiation (>3-fold increase compared with other lysosomal proteins) may also contribute to increased susceptibility [43]. Alternatively, reduced susceptibility to virus-induced apoptosis in Mph versus monocytes (Figure 4) may give rise to an apparent increased infectivity.

Human Mph, like monocytes, exhibit functional plasticity [44], [45], [46], [47]. We have previously described conditions for differentiating monocytes into Mph with pro-inflammatory (Mph1) or anti-inflammatory/regulatory (Mph2) characteristics [26], with Mph2 expressing higher levels of scavenger receptor CD163 and being more proficient phagocytes than Mph1 [26]. Here we report that both Mph types could be readily infected with influenza virus. Mph2 showed a small, but significantly higher permissiveness to Udorn infection than Mph1 in terms of HA expression (∼10%). Yet, Mph1 released more infectious virus than Mph2 (∼1.5-fold for MOI = 2, 8 h p.i), as determined by plaque assays. Further studies with infection dose- and temporal kinetics are required to determine the significance of these observations in terms of inflammatory responses following infection *in vivo*. The polarized Mph1 and Mph2 studied here represent extreme ends of a continuum of phenotypes and their *in vivo* counterparts are currently not known [46], [48]. They likely represent late-phase monocyte/Mph and resident cells present during different phases of the immune response following influenza virus infection, respectively. Our data suggest that Mph are susceptible to influenza virus infection throughout the course of disease. Mph differentiation state in lungs of pathogen-exposed individuals may critically determine disease outcome [29], and people with asthma, a type-2 environment, show enhanced incidence of respiratory infections [49]. Increased prevalence of Mph2-like cells may also render individuals more susceptible to influenza infection.

In both monocytes and Mph, influenza virus infection resulted in virus-dose dependent release of inflammatory cytokines/chemokines. Somewhat surprisingly, Mph1 and Mph2 produced similar cytokine responses following Udorn-infection. This finding is in marked contrast with clearly distinct cytokine profiles of Mph1 and Mph2 exposed to bacterial products [26], [29], [50], sonicated mycobacteria [29], [50], or infection with live bacteria [29]. Influenza may induce similar signaling events in Mph1 and Mph2, but whether this is influenza-specific or a general feature of the response pathway to viruses as opposed to bacteria remains to be determined. It is possible that pattern recognition receptors sensing bacteria are differentially expressed by Mph1 and Mph2, while receptors recognizing influenza virus are expressed similarly. Cytokine production by human monocytes/Mph in cell culture [39], [42], [51], [52] and *in situ* in a lung tissue culture model [53] in response to influenza virus has been reported previously. Of note, with MOIs of 0.5–2 the Poisson distribution predicts that a certain cell percentage will remain uninfected. To date no one has dissected the contribution of the infected versus uninfected cells to cytokine secretion following influenza A virus infection of monocytes/Mph, and we can also not say with certainty that cytokine production results directly from infection, nor whether it is exclusively triggered in infected cells. However, the vast majority of Mph secreting cytokines (Figure 6) express HA (Figure 3). Moreover, we would expect to see different kinetics if there was a significant contribution of uninfected cells to cytokine secretion following Udorn infection (namely, reduced protein levels when the uninfected cell percentage decreases and infected cell percentage increases, while we see the opposite: increased protein release with increased virus dose (Fig. 5) and infection time (Figure 6)). Assuming there is a considerable contribution from infected cells to the cytokine secretion, then future studies might reveal whether and how cells of the Mph lineage override NS1-mediated impairment of anti-influenza immune responses, including blocking the correct processing and export of cellular mRNAs from the nucleus to the cytoplasm [54], [55], [56]. There is a growing body of evidence that certain NS1-mediated countermeasures are influenza-strain specific [57], [58], [59]. Possibly, host cell-specific differences in transcriptional regulation of anti-viral proteins are also a determining factor as to whether or not viral NS1 can counteract the cell's protective responses.

We did not detect IL-33 in supernatants of Udorn-infected human MNC (4 donors) and Mph1/Mph2 cells (6 donors) 8–16 h p.i.. This finding contrasts with increased IL-33 mRNA and protein by CD45^+ve^ cells [60], possibly alveolar Mph [61], in lungs of influenza-infected mice. Influenza strain and/or inter-species differences may account for this apparent discrepancy. In our model, infection beyond 24 h caused excessive apoptosis and experiments were typically performed for 8–16 h. Interestingly, Le Goffic *et al* specifically report a positive association between the IL-33 levels produced by murine and human lung epithelial cell lines and the cytopathic effect of the influenza virus strain, raising the possibility that IL-33 release is associated with virus-induced apoptosis [60].

We found that cells of the Mph lineage exhibited relative resistance to influenza-induced cell death early after infection with MOIs 0.5–2, with apoptosis levels (as assessed by annexin V staining) approximately 10–30%, while the majority of cells expressed HA at the cell surface. Similarly, in Udorn-infected MDCK and epithelial A549 cells, HA-expression precedes onset of apoptosis at 8 h p.i. (MOI = 2,) with 72% and 61% of cells expressing HA, respectively, whilst apoptosis was 25% and 12% in these populations (data not shown), corroborating our previous findings [62]. Whether this ‘delay’ in Mph apoptosis compared to HA expression is due to active strategies by the host cell to delay apoptosis and allow engagement of various anti-viral mechanisms, or whether the virus acts to delay host cell apoptosis to maximise viral protein synthesis (Figure S1) and production of infectious virions, remains to be determined. Apoptotic cell removal by cells of the Mph lineage likely contributes to resolving inflammation following influenza infection [63], likely through suppression of pro-inflammatory cytokine production, induction of anti-inflammatory mediators, and promotion of tissue repair [22], [26], [64]. We here report that influenza infection enhanced the uptake of apoptotic epithelial cells and neutrophils (∼4-fold) by poorly phagocytic Mph1. The phagocytic capacity of Mph2, however, was not affected following influenza infection, possibly because the phagocytic capacity of Mph2 was already maximal. The observed increase in phagocytosis was not mediated by a soluble product, as shown by supernatant transfer experiments, so the functional alteration likely represents a direct consequence of virus infection rather than a secondary effect via released proteins.

Apoptotic neutrophils were ingested by both HA^+ve^ and HA^−ve^ Mph following infection (Figure 7D), but increasing virus dose augmented cell uptake by HA^+ve^ Mph1 (Figure 7D). Influenza A NS1 binds to and activates host cell phosphoinositide 3-Kinase (PI3K) [62], [65]. Since phagocytic uptake of apoptotic cells by Mph critically depends on PI3K activation [66], it would be interesting to see whether influenza A/Udorn mutant viruses that cannot activate PI3K [62], [65] fail to increase phagocytosis of apoptotic cells by infected Mph. Unlike enhanced phagocytosis by glucocorticoid–treated human Mph [67], influenza-induced phagocytosis increase was independent of IL-10 or Mertk/Protein S pathway. We speculate that expression of viral neuraminidase (NA) contributes to increasing the phagocytic capacity of infected Mph. Indeed, NA treatment of human Mph or apoptotic neutrophils increases uptake of the latter [68], [69], while surface desialylation of Mph with viral NA affects uptake of virus-infected target cells by uninfected murine Mph [70].

Augmenting phagocytosis by recruited inflammatory monocytes and Mph1-like cells during influenza infection may have profound consequences. Failure to suppress influenza-induced cytokines via appropriate apoptotic cell uptake may result in excessive inflammation and lung tissue damage, a suggestion supported by the finding that inhibiting phagocytosis augments lung inflammation and fatality in influenza-infected mice [71]. Understanding the impact of virus-infected apoptotic cells and phagocytes on phagocyte responses in an influenza-infected lung may provide a new angle for the development of therapeutics. Control of apoptotic cell clearance in the inflamed lung may allow redirection of disease outcome and restoration of homeostasis in the respiratory tract as influenza infection progresses.

## Materials and Methods

### Ethics statement

The study was conducted according to the principles expressed in the Declaration of Helsinki, and approved by the Lothian Regional Ethics Committee, UK. Informed written consent was obtained from all volunteers before donating blood.

### Cell preparation

Human peripheral blood was taken from the antecubital vein of healthy volunteers and collected into tubes containing sodium citrate (1% final concentration) to prevent coagulation. Leukocyte populations were then isolated by sedimentation with dextran (500,000 M.wt) followed by discontinuous Percoll™ gradient centrifugation, as reported previously [72]. Macrophage subsets were generated from the mononuclear cell fraction (MNC) as described [26]. In short, monocytes were enriched by anti-CD14 microbeads magnetic cell sorting (Miltenyi Biotec), with a typical purity of 95–98% as assessed by flow cytometry. CD14^+ve^ monocytes were cultured for 6 days in tissue culture plates (Costar) in Iscove's Modified Dulbecco's Medium (IMDM; PAA Laboratories) containing penicillin/streptomycin (PPA Laboratories, 50 U/ml) and 10% heat-inactivated FCS (Biosera), in the presence of 50 U/ml GM-CSF (Peprotech) to generate Mph1, or 25 ng/ml M-CSF (R&D Systems) to generate Mph2. Following stimulation with LPS (E.coli, serotype O127∶B8, Sigma-Aldrich) (10 ng/ml for 24 h) Mph1 cells secreted IL-12 but not IL-10, while Mph2 secreted IL-10 but little IL-12, confirming macrophage polarization [26].

MNC were enriched for monocytes by depleting CD3^+ve^ cells using anti-CD3 microbeads magnetic cell sorting (Miltenyi Biotec), resulting in ∼3-fold increase in the percentage of monocytes present (from 15.9%±1.9 (n = 7) to 49.6%±6 (n = 5)). MNC were maintained in IMDM containing 10% autologous serum and penicillin/streptomycin (PPA Laboratories, 50 U/ml) in Teflon pots (Pierce) (2×10^6^ cells/ml).

Airway epithelial A549 cells and Madin-Darby canine kidney (MDCK) cells were obtained from the European Animal Cell Culture Collection and maintained in Dulbecco's modified Eagle medium (DMEM) (Gibco DMEM/F-12+Glutamax, Invitrogen), supplemented with penicillin/streptomycin (PPA Laboratories, 50 U/ml) and 10% heat-inactivated FCS (Biosera) [62].

### Virus preparation and infection

Influenza A/Udorn/72 virus (Udorn; H3N2 subtype) was prepared by Drs Jackson and Killip, St Andrews University, as published previously [62]. In short, virus was propagated through two passages in MDCK cells overlaid with serum-free DMEM containing 2.5 µg/mL *N*-acetyl trypsin (NAT, Sigma) at 37°C, followed by plaque assay titration on MDCK cells. Viral titres of Udorn stock solution and culture supernatants were determined with plaque assays. MDCK cells were grown to near-confluency in 6 well-plates and infected with 5-fold dilution series of virus in serum-free DMEM at 37°C. After 1 h, the inoculum was removed and cells overlayed with DMEM containing 1% agarose and 2 µg/ml NAT. After 48–72 h cells were fixed for 1 h with 10% buffered formalin (Sigma) and washed with PBS. After agar plug removal, plaques were counted by eye and expressed as Plaque Forming Units (PFU)/ml.

Influenza infection of human MNC and monocytes was performed by infecting 2×10^6^ cells with Udorn or UV-treated virus (0.5 J/cm^2^ for 6 min in a Uvitec cross linker) at indicated MOIs in serum-free IMDM for 1 h at 37°C in Teflon pots, after which the cells were collected by centrifugation (200 g, 3 min). Subsequently, cells were cultured in 1 ml serum-containing medium in Teflon pots. Influenza infection of human monocyte-derived macrophages, A549 cells, MDCK cells and monocytes in experiments to assess a possible effect of cell adherence on infectivity, was performed by incubating 4×10^5^ cells with Udorn, or UV treated virus (0.5 J/cm^2^ for 6 min) at indicated MOIs in serum-free medium for 1 h at 37°C in 24 well tissue culture plates (Costar), after which the inoculum was aspirated and cells cultured in 0.5 ml serum-containing medium. At various times p.i. cell supernatants were stored at −20°C for future analyses, and cells were harvested for flow cytometry (see below). We routinely performed CD14-labelling of the cells after isolation and after infection to confirm the populations we analysed were indeed monocytes (CD14-high expressing cells typically amounted to 89–93%).

To assess the role of caspase activity, cells were incubated with the pan-caspase inhibitor Z-VAD(OMe)-FMK (Bachem) (100 µM) for 30 min prior to infection with Udorn. zVAD was also present in the culture medium (100 µM) during incubation of the cells with virus and was added again after removal of the inoculum for the duration of cell culture (100 µM).

### Flow cytometric analysis

Cells were harvested from Teflon pots (MNC) or detached from plates using trypsin/EDTA (PAA Laboratories) for 20 min at 37°C (macrophages, A549 cells and MDCK cells) or ice-cold EDTA (Gibco; 5 mM) for 20 min on ice (monocytes). All subsequent steps were performed on ice. Cells were incubated for 10 min in PBS containing 50% autologous serum to reduce non-specific antibody binding to FcγR, and virus was inactivated by UV cross-linking. Cells were incubated with FITC- or PE-labelled antibodies or appropriate isotype controls (Becton-Dickinson, UK), diluted in flow buffer (PBS/2% FCS) for 30 min.

To assess surface expression of influenza HA associated with infectivity, cells were incubated with a mouse anti-HA antibody (mouse anti-X31 (H3N2)) or isotype controls, washed twice in flow buffer, and then incubated with a polyclonal goat-anti mouse immunoglobulins (Igs) secondary antibody (APC-conjugated, multiple adsorption igs (Pharmingen, 0.4 µg/ml) or PE-conjugated F(ab′)_2_ igs (DAKO, 1/350)). To assess apoptosis (AxV^+^/PI^−^) and necrosis (AxV^+^/PI^+^) cells were stained with annexin V-APC (Invitrogen) in binding buffer (HBSS containing 2 mM CaCl_2_) for 15 min on ice, followed by addition of propidium iodide (Sigma, 3 µg/ml), added 1 min prior to acquisition.

Data were acquired using a BD FACSCalibur flow cytometer (Becton-Dickinson) using CELLQUEST software and subsequently analysed using FlowJo software (Tree Star).

### Confocal microscopy

Slides were prepared from mock- and Udorn-infected MNC cultured in Teflon pots (1×10^5^ cells/slide) using a Cytocentrifuge (Shandon) at 200 g for 3 min. Cells were fixed with 3.6% formaldehyde in PBS (15 min, RT), treated with 50 mM ammonium chloride in PBS (10 min, RT) to block free formaldehyde groups, permeabilised with 0.5% Triton X-100 in PBS (5 min, RT) and blocked with 3% BSA in PBS (30 min, RT). Cells were stained for 1 h at RT with 50 µl anti-AH (H3N2) (see flow cytometry) (1/1000 in 0.3% BSA/PBS), followed by goat anti-mouse AF488 (Invitrogen, 1/1000 in 0.3% BSA/PBS) plus Hoechst 33342 (Invitrogen, 1 µg/ml in 0.3% BSA/PBS). After washing, cells were mounted in Prolong Gold (Invitrogen), dried O/N and analysed using a Leica SP5C Spectral confocal laser scanning microscope (Leica Microsystems).

### Phagocytosis assay

Human neutrophils were labelled for 30 min with CellTracker™ Green CMFDA (500 ng/ml; Invitrogen) as described [72] and then cultured at 10×10^6^/ml in IMDM containing 10% heat-inactivated FCS and penicillin/streptomycin (50 U/ml) for 20 h at 37°C/5%CO_2_, resulting in spontaneous apoptosis [26].

Mph1 and Mph2 were mock-infected or Udorn-infected for 16 h (MOI = 1) and co-cultured with labelled apoptotic neutrophils in a ratio of 1∶5 in IMDM/10% FCS plus penicillin/streptomycin (50 U/ml). For assessment of phagocytosis of latex microspheres, macrophages were incubated with 1 µm fluorescein-labelled Fluoresbrite particles (Polysciences) (10/20/40 beads per cell). After 1 h at 37°C non-internalised neutrophils or beads were removed by washing. Macrophages were detached with trypsin/EDTA (20 min at 37°C) and virus was then inactivated by UV cross-linking. The percentage of Mph1 and Mph2 capable of phagocytosis was determined by flow cytometry using a FACSCalibur, as described previously [26], [72]. To test the effect of cell supernatant of infected macrophages, culture supernatants of mock- and Udorn-infected Mph1 and Mph2 were harvested 12 h or 14 h p.i., UV-treated, and added to (autologous) untreated Mph1 or Mph2, respectively, and left for 2 h or 4 h until the phagocytosis assay.

To assess a potential role for Mertk, Protein S or IL-10 in phagocytosis of apoptotic cells by influenza-infected macrophages, mock- or Udorn-infected Mph1 and Mph2 were incubated with an anti-Mer blocking antibody (R&D MAB8912; 5 µg/ml), Protein S (Enzyme Research HPS3652L; 4 µg/ml) or recombinant human IL-10 (Peprotech; 20 ng/ml) for 30 minutes before addition of the apoptotic neutrophils. The reagents were also present during the 1 h macrophage-neutrophil co-culture.

### Cytokine detection

Cells were mock-infected or infected with Udorn at indicated MOIs and times, or stimulated with 20 ng/ml LPS (E.coli, serotype O127∶B8, purchased from Sigma-Aldrich) for 20 h, and secreted chemokines and cytokines quantified in UV-irradiated supernatants using ELISAs (IP-10, IL-6, IL-8, IL-10, IL-12p40 and TNFα (optEIA, Beckton-Dickinson) and IL-33 (Legend Max, Biolegend)).

### Western blotting

Macrophages were infected as described above, in 6 well tissue culture plates (2.5×10^5^ cells/well), and proteins extracted by lysing cells 5 min with 150 µl/well M-PER Protein Extraction Reagent (Pierce) containing protease inhibitor cocktail (Sigma, used at 1/100). To assess the role of protein synthesis, cells were incubated with cycloheximide (50 µg/ml; Sigma) for 30 min prior to infection with Udorn. Cycloheximide was also present in the culture medium following incubation of the cells with virus for the duration of cell culture (50 µg/ml). To rule out interference of the pan-caspase inhibitor zVAD with virus attachment, cells were treated with zVAD prior, during and after virus–inoculation, as described above. Lysates (15 µl) were mixed with sample buffer, heat denatured and resolved on 12% Tris-HCl polyacrylamide gels under reducing conditions, followed by transfer onto PVDF membranes (Hybond-P, Amersham) (50V, 1 h). After blocking with 5% Marvel in TBS/0.1% Tween buffer, membranes were incubated with an affinity-purified sheep anti-influenza A NS1 antibody. Following incubation with HPR-conjugated polyclonal rabbit-anti goat Igs secondary antibody (Dako; 1/2000), signal was detected with chemiluminescence (ECL Plus Western Blotting Detection Kit, Amersham) and exposure to Hyper ECL film (Amersham). For subsequent detection of other Influenza A virus proteins (HA, NP and M1) and mammalian β-actin, blots were washed, stripped for 15 min with Pierce Restore WB Stripping buffer (ThermoFisher), blocked for 1 h, and incubated with an anti-Influenza A HA/NP/M1 antibody (sheep anti-X31 (H3N2)) or anti-β-Actin (Sigma, clone AC-15, 1/10,000), followed by HPR-conjugated polyclonal rabbit-anti goat Igs secondary antibody (Dako; 1/2000) or HPR-conjugated polyclonal goat-anti mouse Igs secondary antibody (Dako; 1/2000), respectively. A lysate of Influenza A/Udorn infected A549 cells (16 h, MOI = 5) was used as a positive control.

### Data Analysis

Data are presented as means ± SD, with n = number of independent experiments, or as means ± SD of a representative experiment performed in triplicate. Statistical significance was evaluated using a Student's *t* test.

## Supporting Information

Figure S1 **Influenza infection of human monocyte-derived macrophages results in production of viral proteins.** Influenza infection with infectious, but not UV-inactivated Udorn virus induces production of influenza A proteins NS1 (26 kDa) (**A**) and HA0 (approximately 75 kDa), NP (55 kDa) and M1 (26 kDa) (**B**) in human Mph1 cells, as assessed by Western blotting of cell lysates of mock-infected, UV-inactivated (MOI = 5, 8 h p.i.) and infectious virus-infected cells (MOI = 2 and MOI = 5, 8 h p.i.). None of these proteins were detected in Udorn-infected cells (MOI = 5) treated with protein synthesis inhibitor cycloheximide, while β-Actin was readily detected in all samples (**C**). A lysate of A549 cells infected for 16 h with Udorn virus (MOI = 5) was run as a positive control. Due to the presence of N-acetyl trypsin during infection of these cells, HA was cleaved into HA1 (approximately 55 kDa) and HA2 (approximately 25 kDa), showing as additional 55 kD and 26 kD bands, respectively. Blots are representative examples of 2 independent experiments.
(TIF)
