# Supplementary figures and images for: Influenza Virus A Infection of Human Monocyte and Macrophage Subpopulations Reveals Increased Susceptibility Associated with Cell Differentiation
Source: PLoS One. 2012 Jan 4;7(1):e29443. doi: 10.1371/journal.pone.0029443 (PMC3251590; doi:10.1371/journal.pone.0029443)

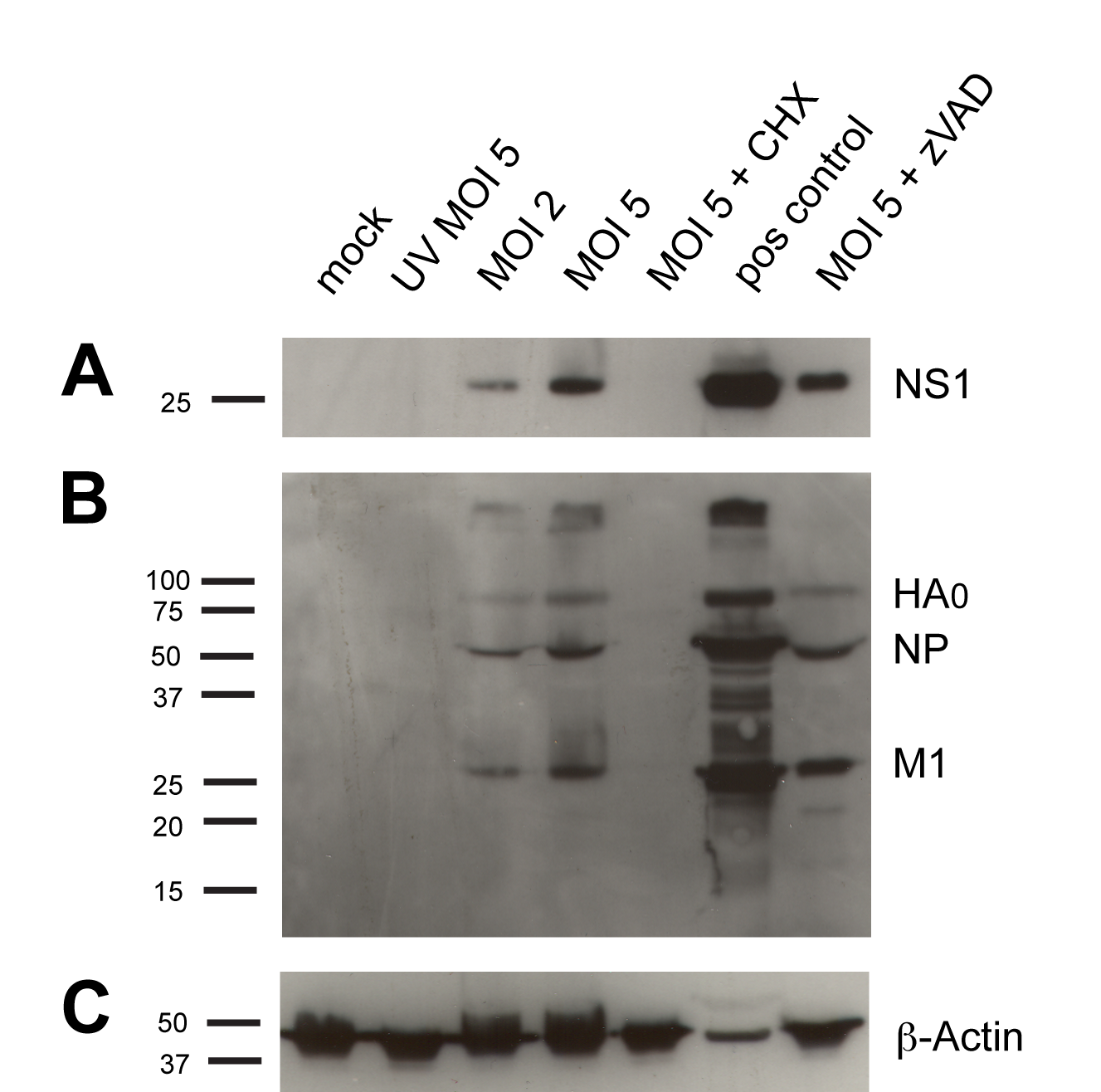

Supplement: Figure S1 — Influenza infection of human monocyte-derived macrophages results in production of viral proteins. Influenza infection with infectious, but not UV-inactivated Udorn virus induces production of influenza A proteins NS1 (26 kDa) (A) and HA0 (approximately 75 kDa), NP (55 kDa) and M1 (26 kDa) (B) in human Mph1 cells, as assessed by Western blotting of cell lysates of mock-infected, UV-inactivated (MOI = 5, 8 h p.i.) and infectious virus-infected cells (MOI = 2 and MOI = 5, 8 h p.i.). None of these proteins were detected in Udorn-infected cells (MOI = 5) treated with protein synthesis inhibitor cycloheximide, while β-Actin was readily detected in all samples (C). A lysate of A549 cells infected for 16 h with Udorn virus (MOI = 5) was run as a positive control. Due to the presence of N-acetyl trypsin during infection of these cells, HA was cleaved into HA1 (approximately 55 kDa) and HA2 (approximately 25 kDa), showing as additional 55 kD and 26 kD bands, respectively. Blots are representative examples of 2 independent experiments. (TIF) [file pone.0029443.s001.tif]
